# Calculated Terahertz Spectra of Glycine Oligopeptide Solutions Confined in Carbon Nanotubes

**DOI:** 10.3390/polym11020385

**Published:** 2019-02-25

**Authors:** Dongxiong Ling, Mingkun Zhang, Jianxun Song, Dongshan Wei

**Affiliations:** 1 School of Electrical Engineering and Intelligentization, Dongguan University of Technology, Dongguan 523808, Guangdong, China; lingdx@dgut.edu.cn (D.L.); songjx@dgut.edu.cn (J.S.); 2 Chongqing Engineering Research Center of High-Resolution and 3D Dynamic Imaging Technology, Chongqing Institute of Green and Intelligent Technology, Chinese Academy of Sciences, Chongqing 400714, China; zhangmk@cigit.ac.cn

**Keywords:** terahertz spectroscopy, oligopeptide, carbon nanotube, confinement, dielectric constant, molecular dynamics

## Abstract

To reduce the intense terahertz (THz) wave absorption of water and increase the signal-to-noise ratio, the THz spectroscopy detection of biomolecules usually operates using the nanofluidic channel technologies in practice. The effects of confinement due to the existence of nanofluidic channels on the conformation and dynamics of biomolecules are well known. However, studies of confinement effects on the THz spectra of biomolecules are still not clear. In this work, extensive all-atom molecular dynamics simulations are performed to investigate the THz spectra of the glycine oligopeptide solutions in free and confined environments. THz spectra of the oligopeptide solutions confined in carbon nanotubes (CNTs) with different radii are calculated and compared. Results indicate that with the increase of the degree of confinement (the reverse of the radius of CNT), the THz absorption coefficient decreases monotonically. By analyzing the diffusion coefficient and dielectric relaxation dynamics, the hydrogen bond life, and the vibration density of the state of the water molecules in free solution and in CNTs, we conclude that the confinement effects on the THz spectra of biomolecule solutions are mainly to slow down the dynamics of water molecules and hence to reduce the THz absorption of the whole solution in confined environments.

## 1. Introduction

Terahertz (THz) wave is one of the electromagnetic radiations lying between the millimeter and far-infrared waves with a frequency range from 0.1 to 10 THz [1]. Since the energy level of the biomolecular low-frequency motions including vibration, rotation, and translation of the molecular skeleton largely falls into the energy level of a THz wave, a THz wave can well provide vibration information of these motions and thereby be capable of being a unique spectroscopy technology to detect biomolecules without the requirement of markers [2,3]. When the THz wave is used to detect biomolecules, the radiation energy is relatively low with a value of ~4 meV per photon, which will hardly cause ionizing damage to the structure of biomolecules. Meanwhile, there are several notable features of the biomolecular THz spectroscopy that differentiate it from traditional spectroscopies. Firstly, the spectral intensity of a THz wave is generally 1~2 orders of magnitude lower than those of the infrared and Raman, which makes testing THz signals difficult. Secondly, absorption peaks are not related to specific functional groups, but with the all-structure motions of biomolecules. Thirdly, there is no THz spectroscopy database, which makes the quantitative spectral analysis difficult. Fourthly, besides the intrinsic structural properties of the biomolecule, the THz spectrum is more related to the interaction between the biomolecule itself and its surrounding water molecules. Due to these features, THz spectroscopy detection of biomolecules in aqueous solution is full of challenges. The characteristic fingerprint signals of biomolecules are not prominent or can, in some cases, not be observed [4,5,6,7] due to the strong absorption of water in solution.

With the emerging applications of THz spectroscopy in biomedicine, THz spectroscopy detection of biomolecules is essential as biomolecules are the basic elements of biological cells and tissues. Since all biomolecules in vivo are almost in aqueous environments, THz spectroscopy detection of biomolecules in solution has become a hot topic in the last decade. To overcome the strong absorption of water and improve the detection sensitivity, micro/nano-channels are widely utilized for THz spectroscopy measurements of biomolecular solutions. George et al. [8] found the microchannel device could improve the THz spectral sensitivity of the bovine serum albumin (BSA) protein solution and the detected concentration of BSA solution could be as low as 1 pM. Brown et al. [9,10] measured the THz spectrum of RNA molecules in solution using a 600-nm-wide and 500-nm-deep SiO_2_ nanochannel array and found the signal-to-noise ratio was greatly promoted and the spectral resolution improved to 10 GHz. Xia et al. [11] anticipated the potential applications of nanochannel biochips applied in THz spectroscopy detection in their review article. Moreover, the nanochannel chips have prominent advantages in manipulating the conformation [12], the motion, and the interaction process [13] of biomolecules, which will provide the feasibility of THz detection on a single-molecule level. With the continuous upgrade of nano-manufacturing technologies, e.g., the electron beam lithography, nanoimprint, and focus ion beam, more and more nanochannels will be employed for THz spectroscopy detection of biomolecules. 

Despite the above progress on applying nanochannels to enhance the THz detection signals and to improve the measurement accuracy and sensitivity, the question how the nanochannel will influence the THz spectra of biomolecules has not attracted attention. Confinement will occur when the motion of polymers is restricted in a space with a size comparable to the hydrodynamic radius of the polymer due to the conformation entropy reduction of the polymer according to the prediction of the classical theories from Odijk [14] and de Gennes [15] and the experimental observation [16]. Confinement universally exists in the biological system, for example, the cell is a natural confinement environment for DNA and protein molecules. Confinement effects on the conformation [17], mechanical properties [18], diffusion and folding dynamics [19,20,21,22,23], and hydrogen-bond interaction [24] of biomolecules have been widely investigated, and the effects of the molecular conformation [25,26,27,28], hydrogen-bond interaction [29,30], and hydration dynamics [7,31,32,33] on the THz spectra of biomolecules have also been explored in theory and experiment. However, the confinement effects on the THz spectra of biomolecules and the underlying mechanism remain unknown to date.

Here, we present all-atom molecular dynamics (MD) simulations to investigate the confinement effects on the THz spectrum of a glycine oligopeptide solution confined in carbon nanotubes (CNTs) with different radii. By analyzing the diffusion coefficient, the dielectric relaxation time, hydrogen bond lifetime, and the vibration density of state of the water molecules in the CNT, the confinement effects on the THz spectra of biomolecules are discussed in detail. The rest of this paper is organized as follows. In Section 2, we will describe the simulation model and method. In Section 3, we will present and discuss the simulation results of the THz spectra of the oligopeptide solution confined in CNTs. Finally, some key conclusions of this work will be summarized in Section 4. 

## 2. Model and Method

### 2.1. The Free and Confined Gly23 Solution Systems

The glycine oligopeptide Gly23 (ACE-GLY_23_-NME) is chosen as the model biomolecule due to its simple structure and good solubility in aqueous solution. The TIP3P model is used to represent water molecules. Considering the radius of gyration of the Gly23 molecule in solution, six uncapped zigzag single-wall carbon nanotubes of (18,0), (20,0), (23,0), (25,0), (30,0), and (35,0) with different radii of 7.1, 7.9, 9.1, 9.9, 11.9, and 13.9 Å, respectively, and the same length of 20 repeat units are built to construct the confined systems. The interaction between the Gly23 and water molecules is described by the Amber ff03 force field [34] which is widely used for protein simulations. The CNT is modeled by our recently developed PPBE-G force field [35], a simple and highly efficient molecular force field for graphene and CNT [36,37]. The C atoms in CNT interacting with Gly23 and water molecules are modeled as the C in -CH2- of the main chain of a protein described by the Amber ff03 force field. All simulations are performed using the DL_POLY 2.20 [38] software.

**Figure 1:**
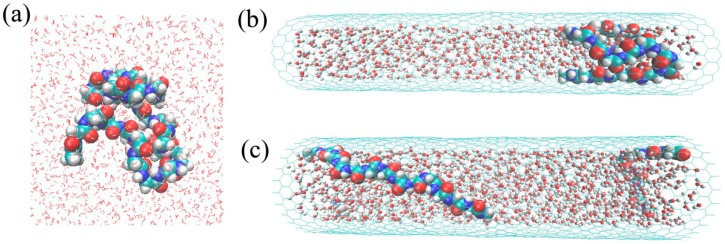
Snapshots of Gly23 solutions in free solution (**a**), CNT (18,0) (**b**), and CNT (25,0) (**c**).

A linear Gly23 oligopeptide was first generated and put into a CNT and simulated for 0.1 ns in a vacuum at 500 K. When running the MD simulation in a vacuum, the center of the biomolecule was tethered at the center of the CNT with a spring force of 100.0 Kcal/mol·Å^−2^ to avoid its escape from the CNT. Then, the CNT and the encapsulated oligopeptide chain were solvated in a periodic water box. Initial sizes of the solvation water boxes were set to 32 × 32 × 130, 34 × 34 × 130, 38 × 38 × 130, 42 × 42 × 130, 46 × 46 × 130, and 50 × 50 × 130 Å^3^, respectively, for different CNTs. After a 5-ns NPT simulation at a pressure of 1 bar and at the temperature of 298.15 K for the solvated CNT systems, the sizes of the equilibrated simulation boxes become 29.6 × 29.6 × 120.1, 33.2 × 33.2 × 119.9, 33.3 × 33.3 × 120.2, 37.2 × 37.2 × 111.6, 39.0 × 39.0 × 111.3, and 44.6 × 44.6 × 111.6 Å^3^, respectively, and the average numbers of water molecules encapsulated in CNTs are 189, 268, 411, 523, 841, and 1230, respectively, with a standard deviation of less than 2% in each case. Subsequently, the CNTs and the encapsulated Gly23 and water molecules were extracted from the above boxes, and then, were aligned along the *z* axis in new periodic boxes with a length in the *z* direction equal to the length of the nanotubes, as shown in Figure 1. The new simulation boxes have the sizes of 30.0 × 30.0 × 85.6, 32.0 × 32.0 × 85.6, 34.0 × 34.0 × 85.6, 36.0 × 36.0 × 85.6, 40.0 × 40.0 × 85.6, and 44.0 × 44.0 × 85.6 Å^3^, respectively, to guarantee that there are vacuum layers of at least 15 Å along the radial *x* and *y* directions. Lastly, a 50-ns NVT simulation at 298.15 K with a timestep of 1 fs was performed for each system. During the NVT simulation, the tethering of a Gly23 chain in the CNT was released. The trajectory and velocity of each atom from the last 10-ns NVT simulation were saved every 0.1 ps for data analysis. For comparison, the Gly23 free solution system comprising a single Gly23 molecule and 1952 TIP3P water molecules in a periodic simulation box with a size of 36.5 × 36.5 × 45.6 Å^3^ was also simulated in the NVT ensemble at 298.15 K for 20 ns and the last 10-ns simulation data were also saved every 0.1 ps for data analysis.

### 2.2. Radius of Gyration and Diffusion Coefficient

To investigate the confinement effect on the Gly23 molecule, the radius of the gyration *R_g_* of the Gly23 chain in the CNT is calculated as:

(1) $$R_{g}=\sqrt{\sum_{i=1}^{N}m_{i}\left[{\left(r_{i}-r_{cm}\right)}^{2}\right]/\sum_{i=1}^{N}m_{i}},$$

where *i* is the atom index, *N* is the total atom number of the Gly23 chain, *m_i_* is the atom mass, **r_i_** is the atom position vector, and **r_cm_** is the center of the mass of the Gly23 chain.

To check the confinement effect on the dynamics of the water molecules, the diffusion coefficient of the water is calculated via the slope of the mean square displacement using the Einstein relation for diffusional motion in three dimensions,

(2) $$D=\underset{t\to \infty }{\lim}\frac{\langle {\left|r(t)-r(0)\right|}^{2}\rangle }{6t}$$

where **r**(t) denotes the position of the oxygen atom in water at time *t* and 〈…〉 represents the average over all water molecules and ensembles.

### 2.3. THz Absorption Spectroscopy

The most interesting optical parameter is the absorption coefficient which can be compared with experiment. In this work, we first calculate the imaginary part of the dielectric constant according to the following equation [7]:

(3) $$\varepsilon^{\prime \prime }\left(\omega \right)=\frac{1}{6\varepsilon_{0}V}\;\frac{2\pi \omega }{k_{B}T}\;\int_{-\infty }^{+\infty }dt\langle M\left(t\right).M\left(0\right)\rangle \exp\left(-i2\pi \omega t\right),$$

where **M**(*t*) is the total dipole moment of the Gly23 solution confined in the CNT including the contributions from Gly23 and water molecules, *V* is the volume of the CNT, *T* is the simulation temperature, *k*_B_ is the Boltzmann constant, *ε*_0_ is the vacuum dielectric constant, *ω* is the frequency as the input variable in calculations with a usual frequency range of 0–3.0 THz for most THz spectrometers, and 〈M(t).M(0)〉 represents the time correlation function of the dipole moment. Once obtaining $\;\varepsilon^{\prime \prime }\left(\omega \right)$, the real part of the dielectric constant can be calculated according the Kramers–Kronig relation,

(4) $$\varepsilon^{\prime }\left(\omega \right)=1+\frac{2}{\pi }\;P\int_{0}^{+\infty }ds\frac{s\varepsilon^{\prime \prime }\left(s\right)}{s^{2}-\omega^{2}}$$

where *P* stands for the principal value of the integral. Since the dielectric constant can be expressed as $\varepsilon \left(\omega \right)=\varepsilon^{\prime }\left(\omega \right)-i\varepsilon^{\prime \prime }\left(\omega \right)={\left[n\left(\omega \right)+ik\left(\omega \right)\right]}^{2}$, with the connection 4πωk(ω)=cα(ω), where *c* is the light speed, n(ω) is the refractive index, and k(ω) is the extinction coefficient, the absorption coefficient α(ω) can be obtained as,

(5) $$\alpha \left(\omega \right)=\frac{2\pi \omega }{c}\sqrt{2\left[\sqrt{\varepsilon^{\prime 2}\left(\omega \right)+\varepsilon^{\prime \prime 2}\left(\omega \right)}-\varepsilon^{\prime }\left(\omega \right)\right]}.$$

## 3. Results and Discussion

### 3.1. Confinement Effects on the Size of Gly23 Oligopeptide and the Water Diffusion Dynamics

**Figure 2:**
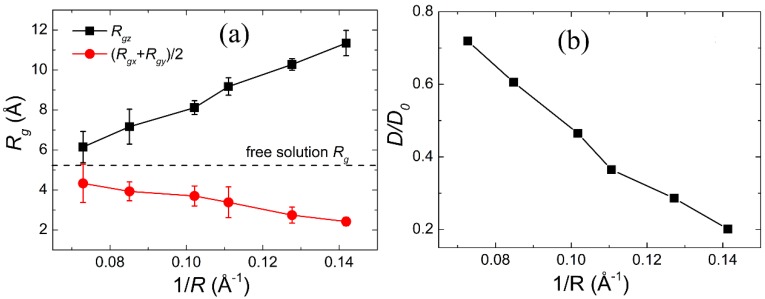
Variations of the radii of gyration of Gly23 in the axial and the radial directions (**a**) and the relative diffusion coefficient of water molecules with the degree of confinement (**b**), where *D*_0_ is the diffusion coefficient of oxygen atoms in bulk water.

As shown in Figure 2a, with the increase of the degree of confinement, 1/*R*, defined as the reverse of the radius of the CNT, *R_g_* of the Gly23 along the radial directions (*R_gx_* or *R_gy_*) has an obvious decrease and *R_g_* along the axial direction (*R_gz_*) almost linearly increases, while the diffusion coefficient of water molecules prominently decreases in comparison with that in the free solution, as shown in Figure 2b. These results are consistent with many previous studies regarding the polymer [39,40] and biomolecules [41,42] in confined environments. These results indicate the CNT implements confinement effects on the Gly23 oligopeptide and water molecules.

### 3.2. Confinement Effect on the Terahertz Absorption Spectrum of Gly23

As discussed in the above section, since the size of the oligopeptide and the dynamics of water molecules are influenced due to confinement, the THz absorption spectrum of the oligopeptide solution in CNT should be different from that in the free oligopeptide solution. According to Equations (3)–(5), the THz absorption spectrum of the oligopeptide solution can be obtained via a series of calculations. To accurately calculate the absorption coefficient, it is key to ensure the convergence of the correlation function of the dipole moment. Here, we evenly divide the last 10-ns NVT trajectory data into 20 independent blocks and calculate the averaged correlation function of the dipole moment over the 20 blocks. For each block, there are 5000 trajectory frames with a time interval of 0.1 ps, which will guarantee the generated frequency spectrum via Fourier transform with a frequency resolution of 2 GHz and a frequency range up to 5 THz.

**Figure 3:**
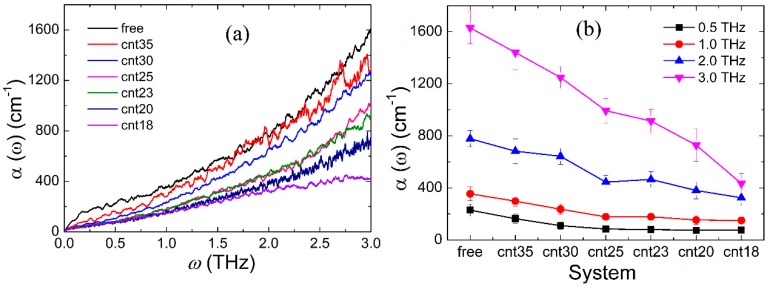
(**a**) THz absorption spectra of Gly23 solutions in different systems; (**b**) variations of THz spectral intensity with simulation systems at different THz frequencies.

From Figure 3a, we can see that the absorption coefficient of the Gly23 solution increases with the increase of the frequency and there is no prominent fingerprint signal; observations which are in good agreement with the THz spectra of most biomolecular solutions [32,43,44,45]. In Figure 3b, it can be seen that the absorption coefficient of the Gly23 solution decreases with the increase of the degree of confinement at different frequencies. As we know from the above analysis, with the increase of the degree of confinement, the diffusion motions of water molecules are more seriously constrained and gradually attenuated, which results in a pronounced decrease in the THz absorption coefficient.

**Figure 4:**
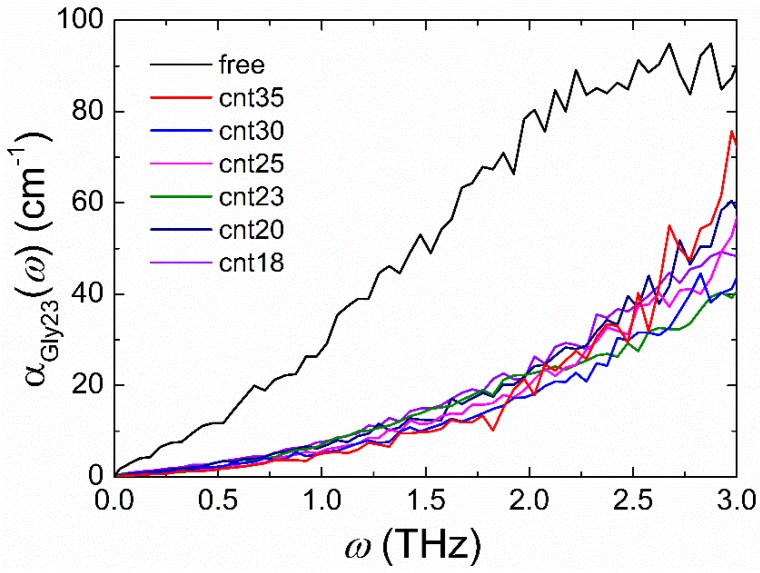
THz absorption spectra of the Gly23 molecule in different systems.

To further look into the confinement effect on the THz spectrum of the Gly23 solution, the variation of the THz absorption coefficient of Gly23 molecule ($\alpha_{\mathrm{Gly}23}$) with the degree of confinement is also investigated. When only the dipole moment of the Gly23 molecule is considered, not including that of the water molecules, the THz absorption coefficients of Gly23 in free solution and confined environments can be approximately calculated in a similar way. It is worth noting that in these cases the volumes in Equation (3) are not the CNT or the periodic simulation box volume but the occupied volume of the Gly23 molecule. Here, we use the excluded volume of the Gly23 molecule, *V_ex_*, (*V_ex_* = 4/3π*R_e_*^3^, where *R_e_* is the end-to-end distance of the Gly23 molecule [46]) to approximately represent the volume in Equation (3) for Gly23 molecules in free solution and different confined environments. Figure 4 shows the frequency dependence of $\alpha_{\mathrm{Gly}23}$ in different environments. Two remarkable features can be observed from this figure. Firstly, in comparison with Figure 3a, the THz absorption coefficients of the Gly23 molecule are an order of magnitude less than those of the corresponding Gly23 solutions in the same environment. This is because the absorption coefficient of the biomolecular solution receives the greatest contribution from water and the absorption of water is one order of magnitude greater than that of most biomolecules. Secondly, $\alpha_{\mathrm{Gly}23}$ of the free solution is prominently larger than those of the confined environments. The decrease of $\alpha_{\mathrm{Gly}23}$ in confined environments probably results from the conformation change of the Gly23 oligopeptide from a flexible coil to a rigid rod, which coincides with our previous theoretical study [26] and the experimental findings [28,47]. While, with the increase of the degree of confinement, the THz absorption coefficients of Gly23 changes slightly. The insignificant change of $\alpha_{\mathrm{Gly}23}$ indicates that there is no further conformation transition of Gly23 occurring under the present confined environments.

Since the decrease of the THz absorption of the Gly23 solution is much more prominent than that of the Gly23 molecule, the THz absorption change of the water molecules in confined environments has the largest contribution to that of the whole solution. Therefore, more attention needs to be paid to the confinement effect on the water molecules. Subsequently, the dielectric relaxation dynamics, the hydrogen bond dynamics, and the vibration density of state of water molecules are analyzed in detail below.

### 3.3. Dielectric Relaxation Dynamics of Water

To determine the hydrogen bond dynamics of the water molecules, the dielectric constants ($\varepsilon^{\prime }$ and $\varepsilon^{\prime \prime }$) of the free and confined Gly23 solutions obtained according to Equations (3) and (4) are analyzed. The Debye model, which describes the dynamics in terms of collective, diffusive, and reorientational motions and has extensively been used for pure liquids and liquid mixtures [48,49,50], can be used to establish the relation between the complex dielectric constant of the solution and the relaxation dynamics of the water molecules by fitting the following equation [51]:

(6) $$\varepsilon \left(2\pi \omega \right)=\epsilon^{\prime }\left(2\pi \omega \right)+i\epsilon^{\prime \prime }\left(2\pi \omega \right)=\frac{\epsilon_{1}-\epsilon_{2}}{1+i2\pi \omega \tau_{1}}+\frac{\epsilon_{2}-\epsilon_{\infty }}{1+i2\pi \omega \tau_{2}}+\epsilon_{\infty },$$

where $\varepsilon_{1}$ is the static permittivity, $\varepsilon_{2}$ is the intermediate frequency dielectric constant.$\;\varepsilon_{1}-\varepsilon_{2}$ and $\varepsilon_{2}-\varepsilon_{\infty }$ are the amplitudes of the two individual processes, respectively, while $\varepsilon_{\infty }$ is the permittivity at an infinitely high frequency. $\tau_{1}$ is the dielectric relaxation time of the slow process, which is assigned to the cooperative relaxation of the hydrogen-bond network of water [49,52], while $\tau_{2}$ is the dielectric relaxation time of the fast process, which is usually attributed to the fast reorientation of a few “free” water molecules in solution [53].

**Figure 5:**
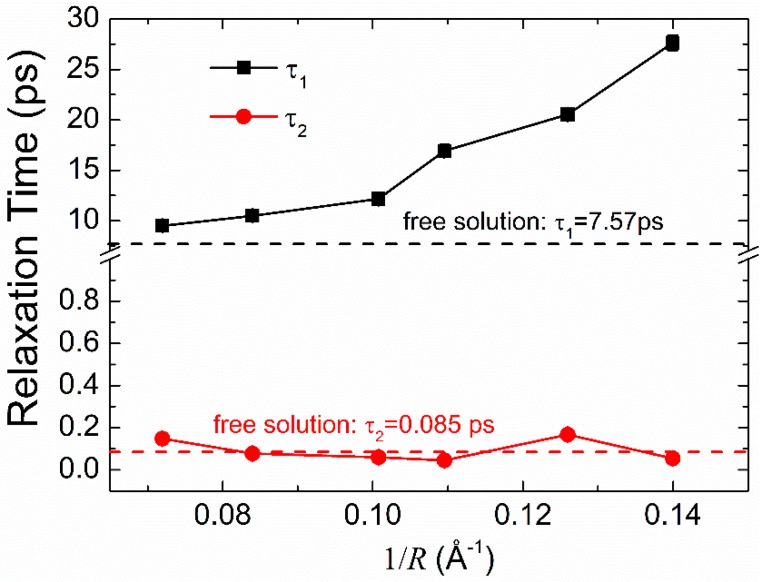
Dielectric relaxation times *τ*_1_ and *τ*_2_ of water molecules.

From Figure 5, we can see that $\tau_{1}$ clearly increases with the increase of the degree of confinement, indicating the retardation of the long-range hydrogen bond interaction due to the confinement effect. While $\tau_{2}$ almost doesn’t change with the increase of the degree of confinement, implying the reorientation dynamics of the individual mobile water are not affected by the confinement effect. These findings are in good agreement with those observed by Qi et al. [54] in their study of water confined in CNTs. Comparing the relaxation times $\tau_{1}$ and $\tau_{2}$ with the results of Qi et al., we find the inclusion of the Gly23 oligopeptide further increases the relaxation time of the long-range hydrogen bond interaction and the reorientation motion of the individual water also becomes slower.

As we know, the retardation of the long-range hydrogen bond interaction will lead to the reduction of the absorption coefficient of water molecules, which will result in the reduction of the absorption coefficient of the whole solution since the absorption coefficient of water is much larger than that of the biomolecule. This may provide a good interpretation of the mechanism of the confinement effect on the THz spectra of the biomolecular solution.

### 3.4. Hydrogen Bond Dynamics

To further explain the variation of the absorption coefficient with the degree of confinement, the dynamics of the hydrogen bonds formed between water molecules are explored.

Hydrogen bonds formed between water molecules are counted and their intermittent rearrangement dynamics are characterized by the hydrogen bond time auto-correlation function as follows [47,55]:

(7) $$C_{HB}\left(t\right)=\frac{\langle h\left(0\right)h\left(t\right)\rangle }{\langle h^{2}\left(0\right)\rangle },$$

where the operator *h*(*t*) gives 1 if a given hydrogen bond is intact at time *t* and 0 otherwise. Brackets denote the ensemble average. Here, the hydrogen bond is defined with a maximum donor–acceptor distance of 3.5 Å and a minimum donor–H–acceptor angle of 135°, which is a regular definition in MD software.

**Figure 6:**
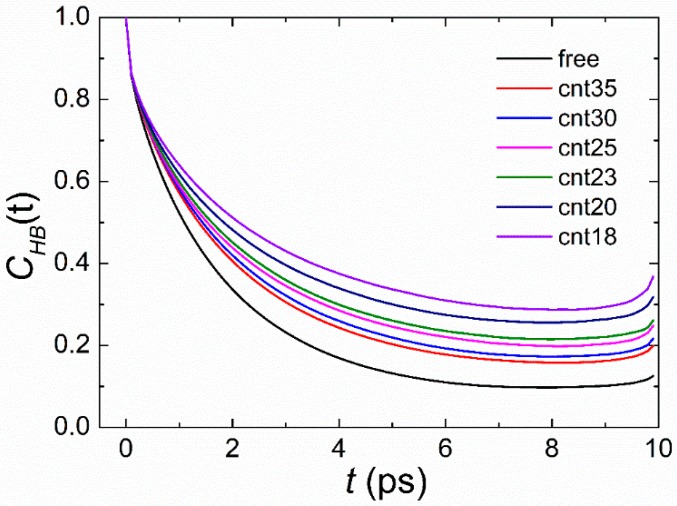
Decay of *C_HB_*(*t*) for different systems.

From Figure 6, it can be seen that *C_HB_*(*t*) decays more slowly with the decrease of the radius of the CNT, which is in good agreement with the previous finding that *C_HB_*(*t*) exhibits slower decay for water molecules confined in the smaller diameter pore [56]. The retarded hydrogen bond dynamics of the water molecules around the oligopeptide can be explained by the imposed steric constraints on the water due to the existence of the wall of the nanotube. Because of the retardation of the hydrogen bond dynamics of the water molecules, the THz absorption coefficient of the oligopeptide solution will decrease, which agrees well with our simulation results in Section 3.2.

### 3.5. Vibration Density of States

To estimate the absorption contribution from water molecules, the vibration density of states (VDOS) of water molecules in CNT are investigated. Here, VDOS was obtained via the Fourier transform of time auto-correlation functions of the atomic velocity **v** [27,47,57,58],

(8) $$\mathrm{VDOS}\left(\omega \right)\sim \int_{0}^{\infty }dtexp\left(i\omega t\right)\langle v\left(0\right).v\left(t\right)\rangle ,$$

Here, the exact prefactor is omitted to yield the VDOS in arbitrary units. This is feasible because this quantity is not directly compared to experiments and only the relative intensities and the frequency shifts are considered. The velocities of the oxygen atoms are chosen for the VDOS calculation because they dominate the low-frequency vibrational spectrum below 200 cm^−1^, while the hydrogen atoms contribute mainly to frequencies above 400 cm^−1^ [57].

**Figure 7:**
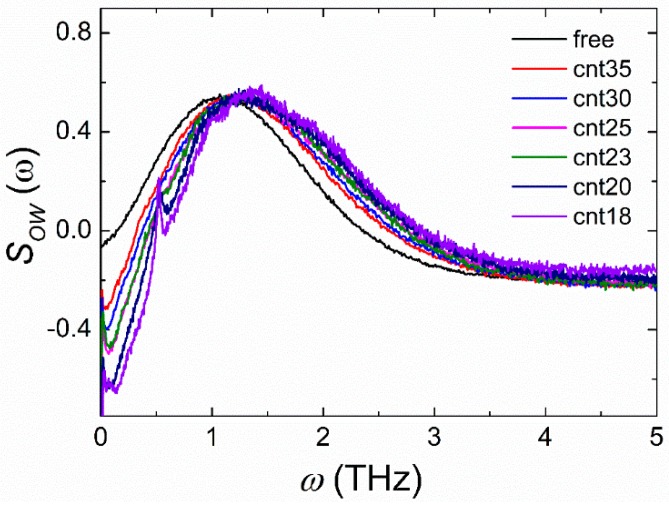
The vibration density of states (VDOS) of oxygen atoms in water molecules for different systems.

Our calculations revealed that, compared to water molecules in free solution, these bands are uniformly blue-shifted and the extent of the shifts is more pronounced for the more confined systems, as shown in Figure 7. These blue-shifts correlate excellently with the slow structural relaxation of the hydrogen bonds, indicating there is a strong caging effect on the dynamics of the water molecules. They were also usually observed for water molecules in the hydration shells of DNA and protein [27,29,47,57], or in the confined pore [56]. It is worth noting that one small sharp peak appears for the most confined two systems of cnt18 and cnt23 at the frequency of around 0.5 THz. The appearance of the small peak denotes a solid-state behavior may exist, indicating the water molecules are closely confined in these two systems. Therefore, from the VDOS analysis, it is further confirmed that the confinement will result in the reduction of the absorption coefficient of the biomolecular solution.

## 4. Conclusions

In this work, the Gly23 oligopeptide in aqueous solution confined in carbon nanotubes with different radii was simulated and the confinement effects on the terahertz spectra of the Gly23 solution were explored. With the increase of the degree of confinement, the THz spectral intensity of the biomolecular solution decreases notably, while the THz spectral intensity of the biomolecule itself changes slightly. Since water molecules usually contribute greatly to the THz absorption of the biomolecular solution, we interpreted the reason for the decrease of the THz spectral intensity according to the diffusion dynamics, the dielectric relaxation dynamics, the hydrogen bond lifetime, and the vibration density of states of water molecules in confined environments. Our simulation and calculation results revealed that the retarded hydrogen bond dynamics of water molecules due to the steric confinement from the wall of the CNT resulted in the decrease of the THz spectral intensity of the Gly23 solution. The findings in this work will help to explore the water dynamics in the nano-confined environment and will guide THz spectroscopy detection of biomolecules using nanofluidic channels in practice.
